# Anxiety, Post–COVID-19 Syndrome-Related Depression, and Suicidal Thoughts and Behaviors in COVID-19 Survivors: Cross-sectional Study

**DOI:** 10.2196/36656

**Published:** 2022-10-25

**Authors:** Sean F Woodward, Sumra Bari, Nicole Vike, Shamal Lalvani, Khrystyna Stetsiv, Byoung Woo Kim, Leandros Stefanopoulos, Nicos Maglaveras, Hans Breiter, Aggelos K Katsaggelos

**Affiliations:** 1 Department of Psychiatry and Behavioral Sciences Northwestern University Chicago, IL United States; 2 Feinberg School of Medicine Northwestern University Chicago, IL United States; 3 Department of Electrical and Computer Engineering Northwestern University Evanston, IL United States; 4 Laboratory of Medical Informatics Aristotle University of Thessaloniki Thessaloniki Greece; 5 Laboratory of Neuroimaging and Genetics Department of Psychiatry Massachusetts General Hospital and Harvard University Boston, MA United States; 6 Department of Computer Science Northwestern University Evanston, IL United States; 7 Department of Radiology Northwestern University Chicago, IL United States

**Keywords:** COVID-19, post–COVID-19 syndrome, suicidality, depression, Patient Health Questionnaire-9, PHQ-9, State Trait Anxiety Index, STAI

## Abstract

**Background:**

Although the mental health impacts of COVID-19 on the general population have been well studied, studies of the long-term impacts of COVID-19 on infected individuals are relatively new. To date, depression, anxiety, and neurological symptoms associated with post–COVID-19 syndrome (PCS) have been observed in the months following COVID-19 recovery. Suicidal thoughts and behavior (STB) have also been preliminarily proposed as sequelae of COVID-19.

**Objective:**

We asked 3 questions. First, do participants reporting a history of COVID-19 diagnosis or a close relative having severe COVID-19 symptoms score higher on depression (Patient Health Questionnaire-9 [PHQ-9]) or state anxiety (State Trait Anxiety Index) screens than those who do not? Second, do participants reporting a COVID-19 diagnosis score higher on PCS-related PHQ-9 items? Third, do participants reporting a COVID-19 diagnosis or a close relative having severe COVID-19 symptoms score higher in STB before, during, or after the first year of the pandemic?

**Methods:**

This preliminary study analyzed responses to a COVID-19 and mental health questionnaire obtained from a US population sample, whose data were collected between February 2021 and March 2021. We used the Mann-Whitney *U* test to detect differences in the medians of the total PHQ-9 scores, PHQ-9 component scores, and several STB scores between participants claiming a past clinician diagnosis of COVID-19 and those denying one, as well as between participants claiming severe COVID-19 symptoms in a close relative and those denying them. Where significant differences existed, we created linear regression models to predict the scores based on COVID-19 response as well as demographics to identify potential confounding factors in the Mann-Whitney relationships. Moreover, for STB scores, which corresponded to 5 questions asking about 3 different time intervals (i.e., past 1 year or more, past 1 month to 1 year, and past 1 month), we developed repeated-measures ANOVAs to determine whether scores tended to vary over time.

**Results:**

We found greater total depression (PHQ-9) and state anxiety (State Trait Anxiety Index) scores in those with COVID-19 history than those without (Bonferroni *P*=.001 and Bonferroni *P*=.004) despite a similar history of diagnosed depression and anxiety. Greater scores were noted for a subset of depression symptoms (PHQ-9 items) that overlapped with the symptoms of PCS (all Bonferroni *P*s<.05). Moreover, we found greater overall STB scores in those with COVID-19 history, equally in time windows preceding, during, and proceeding infection (all Bonferroni *P*s<.05).

**Conclusions:**

We confirm previous studies linking depression and anxiety diagnoses to COVID-19 recovery. Moreover, our findings suggest that depression diagnoses associated with COVID-19 history relate to PCS symptoms, and that STB associated with COVID-19 in some cases precede infection.

## Introduction

### Background

More than 60 million COVID-19 cases, directly linked to over 830,000 deaths, have been reported in the United States at the time of this writing [1]. Although our understanding of the disease pathophysiology continues to develop, COVID-19 is largely considered a respiratory and cardiovascular disease caused by infection by the respiratory virus SARS-CoV-2, with highly variable presentation [2]. Symptoms including fatigue, dyspnea, anosmia, and ageusia have been reported to persist for ≥7 months after infection [3-5] and are now attributed to post–COVID-19 syndrome (PCS), otherwise known as long-haul syndrome.

COVID-19 and its pandemic have also been associated with psychological changes; however, most research on this topic has focused on the COVID-19–free population. In this population, general distress [6] as well as increased levels of anxiety, depression [7,8], and posttraumatic stress disorder symptoms [9] have been observed. These symptoms have been specifically attributed to general fears of infection, interpersonal and economic burdens of social distancing measures [10,11], and downstream sleep disturbances [7,12]. Increased levels of suicidal ideation [13,14] and suicide rates [15,16] in the general population have also been noted, although whether suicide rates have truly increased during the COVID-19 pandemic remains debated [17]. Speculative causes for increased suicidal thoughts and behavior (STB) during the pandemic include increased domestic violence [18], social distancing and decreased interpersonal support [13], and specific psychological phenomena exacerbated by the pandemic such as burdensomeness, thwarted belongingness, and stress sensitivity [19].

Studies of the direct psychological and psychiatric impacts of COVID-19 are relatively sparse. Increased levels of depression, anxiety, and posttraumatic stress disorder symptoms have been observed both during COVID-19 hospitalization [20] and up to 3 months after infection [21-23]. One study observed not only increased anxiety and mood disorders but also increased psychotic and substance abuse disorders at 3 and 6 months after COVID-19 infection [24]. Studies on the long-term psychiatric consequences of SARS-CoV-1 infection forewarn that such sequelae may persist for as many as 50 months after infection [25,26]. Moreover, neurological symptoms such as headaches, concentration difficulties, subjective memory loss, and reduced attention span are reported to accompany more common symptoms of PCS [27].

These psychiatric and neurological sequelae have been proposed to fundamentally predispose patients with COVID-19 infection and COVID-19 survivors to increased STB [28,29]. In particular, the literature features several case studies of suicide attempts among hospitalized patients with COVID-19, whose motives may reflect universal experiences of COVID-19 hospitalization [30,31]. However, only a small number of studies have quantitatively addressed STB in patients with COVID-19; one reported increased suicidal ideation in patients 1 month after COVID-19 hospitalization [32], and another characterized suicides in patients testing positive for COVID-19 infection reported in the media [33]. Interestingly, a study of the general population linked increased suicidal ideation with a desire to seek COVID-19 exposure [13], implying a potential reversal of the assumption that COVID-19 precedes an increase in STB.

### Objectives

This study examined responses to a questionnaire digitally distributed to a population sample of 506 adults in early March 2021. The questionnaire included a depression screener (Patient Health Questionnaire-9 [PHQ-9]) [34], a state anxiety screener (State Trait Anxiety Index [STAI]) [35], a custom set of questions about STB experienced in 3 periods (1 month ago, 1-12 months ago, and >12 months ago), a question asking whether the participant ever received a COVID-19 diagnosis, and a question asking whether the participant ever had a friend and/or family member who had severe or fatal COVID-19 symptoms. Data analysis focused on 3 questions. First, assuming a similar prevalence of depression and anxiety history among participants with and without COVID-19 history, we asked whether COVID-19 infection (a personal diagnosis or severe symptomology in a close friend or relative) was linked to—and explicitly causative of—increased depression (PHQ-9) or anxiety (state component of the STAI) screening scores. Second, we asked whether COVID-19 infection was linked to—and implicitly causative of—increases in specific-item PHQ-9 scores and specifically in PCS-related scores (in particular, scores corresponding to fatigue, problems with concentration, psychomotor retardation or agitation, and altered appetite) for patients admitting a COVID-19 diagnosis. Lastly, we asked whether COVID-19 infection was linked to increased STB before or after infection and whether any causality in either direction was suggested. In all cases, we considered confounding factors from demographic information, as well as previous diagnoses of depression and anxiety. This study sought not only to confirm known associations between the experience of COVID-19 and greater depression and anxiety scores but also to expose the drivers of greater depression scores with respect to known PCS symptomology. Moreover, it sought to clarify the effect of COVID-19 on STB in a population reporting pre–COVID-19 STB.

## Methods

### Participant Recruitment and Demographics

Study participants were recruited by Gold Research Inc from multiple vendors. Gold Research’s vendors recruit the emails of willing participants in multiple ways. Some are recruited “by invitation only” from customer databases of large companies in revenue-sharing agreements, some are recruited from social media, some are recruited via direct mail, and others sign up voluntarily to participate in research studies in lieu of monetary or other incentives such as coupons for everyday household purchases. During recruitment, all survey respondents also go through a double opt-in process to indicate the types of research studies they would like to participate in along with providing their profiles on different demographic attributes like age, race, and gender. This information is then used to reflect representation against US Census metrics. In this process, respondents are also asked multiple test questions to screen out those providing random and illogical responses or showing flatline or speeder behavior. Along with having cohort demographics balanced to meet the demographic criteria established by the US Census, Gold Research also oversampled 15% of the sample for mental health conditions.

Gold Research reported that over 50,000 respondents were contacted for questionnaire completion. They estimated that over 37,500 (75%) either did not respond or said no. Of the remaining 12,500 who did click on the survey link, more than 50% did not complete the questionnaire. Of the >6000 who completed the survey, those who did not clear the data integrity assessments were omitted to obtain the final number of completed surveys.

We assessed multiple mental health conditions including depression symptoms and STB. In this study, we focused only on depression and STB. The request for participation stated Gold Research was administering the study on behalf of its client, Northwestern University, to study emotional health (see text at end of this section for detailed instructions in the survey about the solicitation, study description, and opt-in procedures). Participants meeting quality assurance procedures (including completion of the survey) were studied, up to a limit of 500 to 520 participants, resulting in 506 participants in the final cohort, of which 379 (74.9%) met all quality assurance criteria.

Questionnaire responses were digitally collected between the end of February 2021 and the first week of March 2021, approximately 1 year following the official pandemic declaration in the United States (March 11, 2020) [36]. Final filtering for data quality (see the Data Quality Assurance section) reduced the final sample size to 379 questionnaires or participants (Figure S1 in Multimedia Appendix 1). All analyses centered on this subset of 379 participants, which, based on the nature of the databases sampled by Gold Research Inc, as well as the demographic statistics shown below, was determined to be an approximate, random sample of the adult US population. Assessment of location data by state showed that the sample of 379 participants had a broad geographic distribution (Figure S2 in Multimedia Appendix 1). Participants reported age, gender (“What gender do you consider yourself?”), ethnicity (“What ethnicity do you consider yourself?”), annual household income, employment status, years of school, and highest level of education (Table 1).

The final sample comprised 379 participants (age: mean 43, SD 15 years; range 18-70 years) (Table 1). Most participants identified as female (215/379, 56.7%), White (251/379, 66.2%), having an annual household income of US $25,000 to US $50,000 (98/379, 25.9%), employed full-time (161/379, 42.5%), and having completed some college (105/379, 27.7%). The gender, ethnicity, and education percentages approximated US Census Bureau figures at the time of data collection [37].

For initial recruitment, potential participants received the communication displayed in Textbox 1.

If potential participants responded with “Accept,” they were then sent the message in Textbox 2.

The survey would then begin if they pressed “Next.”

The participants were asked questions related to their COVID-19 status. They were asked (1) to report whether they were ever diagnosed with COVID-19 by a medical clinician (variable COVID-DIAGNOSIS: POS/NEG indicates a positive or nondiagnosis), (2) whether they ever received a positive COVID-19 laboratory test (variable COVID-TEST: POS/NEG), and (3) whether a family member or close friend ever experienced serious symptoms or died of COVID-19 (variable COVID-FAMILY: POS/NEG). Because 30 participants were members of both the COVID-DIAGNOSIS POS and COVID-TEST POS groups, which corresponded to 77% (30/39) of the participants of the total COVID-DIAGNOSIS POS group and 73% (30/41) of the participants of the total COVID-TEST POS group, our subsequent analysis featured only the COVID-DIAGNOSIS variable given its collinearity with COVID-TEST. A similar analysis, yielding similar results, for the COVID-TEST variable can be found in Multimedia Appendix 1.

**Table 1:** Participant demographic information with breakdown of questionnaire responses.

Characteristic			Participants^a^
**Age (years)**			
	Count, n (%)	379 (100)	
	Mean (SD); range	43.21 (15.38); 18.0-70.0	
**Gender, n (%)**			
	Male	163 (43)	
	Female	215 (56.7)	
	Other	1 (0.3)	
**Ethnicity, n (%)**			
	White	251 (66.2)	
	African American	52 (13.7)	
	Hispanic	33 (8.7)	
	Asian or Pacific Islander	13 (3.4)	
	Native American or Alaska Native	7 (1.8)	
	Other	3 (0.8)	
	Mixed (≥2 ethnicities)	17 (4.5)	
	Prefer not to answer	3 (0.8)	
**Annual household income^b^ (US $), n (%)**			
	<25,000	88 (23.2)	
	25,000-50,000	98 (25.9)	
	50,000-75,000	73 (19.3)	
	75,000-100,000	48 (12.7)	
	100,000-150,000	38 (10)	
	150,000-300,000	26 (6.9)	
	>300,000	8 (2.1)	
**Employment status, n (%)**			
	Unemployed	61 (16.1)	
	Full-time	161 (42.5)	
	Part-time	43 (11.3)	
	Self-employed	26 (6.9)	
	More than one job	3 (0.3)	
	Retired	61 (16.1)	
	Other	24 (6.3)	
**Years of school**			
	Count, n (%)	379 (100)	
	Mean (SD); range	13.18 (5.15); 1.0-30.0	
**Highest education level, n (%)**			
	Some high school	7 (1.8)	
	High school graduate	99 (26.1)	
	Some college	105 (27.7)	
	Bachelor’s degree	86 (22.7)	
	Some graduate school	13 (3.4)	
	Graduate degree	30 (7.9)	
	Postgrad or doctorate	39 (10.3)	

^a^Response counts across all participants.

^b^Income and education are considered ordinal variables, and their integer values (prepending the long-form responses in the second column) were used for statistical computations.

**Communication sent to potential participants during initial recruitment.**

Gold Research Inc, a national market research firm, and its client, Northwestern University, request your participation in this study of emotional health. We will be evaluating how different emotions and experiences are connected and may relate to our emotional health. The information you provide will be kept confidential, coded to be anonymous so it cannot be connected back to you and will be used only for research purposes. Researchers will not be able to contact you or restudy you after this survey. We will not share your information with any other third party. We will also not use your information to identify you individually or use your responses to market or sell other services or products to you. As part of this effort, you will not be asked to provide any personal identifiers such as your name, email, phone number, address or social media handles. A unique identifier will be generated for you and each survey participant to enhance privacy. As part of the survey process, we will be able to tell if you completed the survey, but we will not be able to tell which answers were yours. For this study, we are going to ask you some questions about yourself and how much you like or dislike a set of pictures. You may discontinue this study at any time. We appreciate your help with this study, given the serious challenges facing many people regarding emotional health at this time. We thank you in advance.

1. Accept
2. Decline

**Follow-up communication sent to participants.**

Thank you for participating in our survey. All responses during this survey are anonymous and confidential. We will be able to tell if you completed the survey, but we will not be able to tell which answers were yours. In this study, we aim to understand how different emotions and experiences relate to visual processing.

We are going to:

- Ask you some questions about yourself
- Have you rate how much you like or dislike a set of pictures

For this study, your identity is protected and your answers are anonymous and confidential. Press “Next” to proceed.

### Ethics Approval

Participation was offered noting that Gold Research was administering an emotional health questionnaire on behalf of Northwestern University, with the phrasing, “We will be evaluating how different emotions and experiences are connected and may relate to our emotional health.” The complete text about the solicitation, study description, and opt-in procedures can be found in the Participant Recruitment subsection of the Methods section. All participants provided informed consent following oversight by the Northwestern University Institutional Review Board, which reviewed and approved the project (approval number STU00213665). The participants were guaranteed anonymity and confidentiality, and the researchers possessed no protected health information.

### Survey Questions and Scoring

Our survey questionnaire consisted of the PHQ-9, the 20 “state” questions from the STAI Form Y, behavioral neurology and mental health questions including those pertaining to STB from the Massachusetts General Hospital Subjective Question screener of the Phenotype Genotype Project in Addiction and Mood Disorder [38], and questions pertaining to COVID-19 history. A cognitive task related to picture ratings was also included (data not reported here) and incorporated into general quality assurance. The questions pertinent to this study are outlined in Table 2, with a breakdown of responses to ancillary demographic questions given in Table 1. The questionnaire is not outlined in its entirety here.

**Table 2:** Questionnaire items.

Variable^a^	Question preamble	Question^b^	Value space^c^
COVID-TEST	N/A^d^	Have you ever tested positive for COVID-19?	Binary
COVID-DIAGNOSIS	N/A	Have you ever been diagnosed with COVID-19 by a medical clinician?	Binary
COVID-FAMILY	N/A	Has anyone in your family or group of friends had serious symptoms or died of COVID-19?	Binary
PHQ9-1	Over the last two weeks, how often have you been bothered by any of the following problems? 1 (not at all), 2 (several days), 3 (more than half the days), 4 (nearly every day)	Little interest or pleasure in doing things	[0,3]
PHQ9-2	Same as above	Feeling down, hopeless, or depressed	[0,3]
PHQ9-3	Same as above	Trouble falling/staying asleep, sleeping too much	[0,3]
PHQ9-4	Same as above	Feeling tired or having little energy	[0,3]
PHQ9-5	Same as above	Poor appetite or overeating	[0,3]
PHQ9-6	Same as above	Feeling bad about yourself or that you are a failure or have let yourself or your family down	[0,3]
PHQ9-7	Same as above	Trouble concentrating on things, such as reading the newspaper or watching television	[0,3]
PHQ9-8	Same as above	Moving or speaking so slowly that other people could have noticed. Or the opposite, being so fidgety or restless that you have been moving around a lot more than usual	[0,3]
PHQ9-9	Same as above	Thoughts that you would be better off dead or of hurting yourself in some way	[0,3]
PHQ9-SUM	Same as above	Sum of the 9 PHQ-9^e^ scores	[0,27]
STAI-SUM	N/A	Sum of the 20 STAI^f^ Form Y State scores	[0,60]
DEPRESSION-YRS	For how many years have you had the following diagnosis by a medical professional, psychologist, or physician?	Depression?	[0,60]
DEPRESSION-HX	Same as above	Depression?	Binary
ANXIETY-YRS	Same as above	Anxiety disorder?	[0,60]
ANXIETY-HX	Same as above	Anxiety disorder?	Binary
S-PASSIVE-LT	Please rate how often you have experienced each of the following (in the following time periods): 1 (never), 2 (rarely), 3 or 4 (sometimes), 5 or 6 (often), 7 (always)	Wish to go to sleep and not wake up—past 1 year or more	[1,7]
S-PASSIVE-MT	Same as above	Wish to go to sleep and not wake up—past 1 month to 1 year	[1,7]
S-PASSIVE-ST	Same as above	Wish to go to sleep and not wake up—past 1 month	[1,7]
S-ACTIVE-LT	Same as above	Wanting to hurt yourself or take your own life—past 1 year or more	[1,7]
S-ACTIVE-MT	Same as above	Wanting to hurt yourself or take your own life—past 1 month to 1 year	[1,7]
S-ACTIVE-ST	Same as above	Wanting to hurt yourself or take your own life—past 1 month	[1,7]
S-PLAN-LT	Same as above	Having a plan to take your own life—past 1 year or more	[1,7]
S-PLAN-MT	Same as above	Having a plan to take your own life—past 1 month to 1 year	[1,7]
S-PLAN-ST	Same as above	Having a plan to take your own life—past 1 month	[1,7]
S-HISTORY-LT	Same as above	Prior attempts at hurting yourself, or taking your own life—past 1 year or more	[1,7]
S-HISTORY-MT	Same as above	Prior attempts at hurting yourself, or taking your own life—past 1 month to 1 year	[1,7]
S-HISTORY-ST	Same as above	Prior attempts at hurting yourself, or taking your own life—past 1 month	[1,7]
S-SAFETY-LT	Same as above	Having a safety plan for not hurting yourself for when those feelings arise—past 1 year or more	[1,7]
S-SAFETY-MT	Same as above	Having a safety plan for not hurting yourself for when those feelings arise—past 1 month to 1 year	[1,7]
S-SAFETY-ST	Same as above	Having a safety plan for not hurting yourself for when those feelings arise—past 1 month	[1,7]

^a^Designations of the scores or variables obtained.

^b^Corresponding questions as phrased in the questionnaire.

^c^Range of possible responses.

^d^N/A: not applicable.

^e^PHQ-9: Patient Health Questionnaire-9.

^f^STAI: State Trait Anxiety Index.

The PHQ-9 includes 9 questions, as outlined in Table 2 [34]. PHQ-9 is a self-report–based screening tool for depression. A numeric response to a single question can take any integer value from 0 to 4. Across 9 questions, a cumulative score ≥5 is associated with mild depression, and a score ≥10 is associated with moderate depression. For statistical analyses, responses to these questions were pooled to obtain cumulative scores and considered independent scores.

The STAI Form Y includes 20 “state” anxiety questions [35]. This questionnaire subset is a tool for assessing a respondent’s current anxiety in response to a perceived danger, based on self-reporting. A numeric response to a single question can take any integer value from 0 to 3, with a greater cumulative score associated with greater anxiety. We considered only cumulative scores.

Five questions pertaining to current and historic STB were included in the questionnaire; these were adapted from the Massachusetts General Hospital Phenotype Genotype Project [38], with direct antecedents in the *Manual of Psychiatric Emergencies 4th Edition* [39]. These are outlined in their entirety in Table 2. The 5 questions were each repeated 3 times for 3 blocks of time asking how often participants experienced symptoms in the (1) past 1 year or more, (2) past 1 month to 1 year, and (3) past 1 month on a 1 to 7 Likert scale (1=never, 2-3=rarely, 4-5=sometimes, 6=often, and 7=always). Responses to these questions were considered independent and were not pooled to obtain cumulative scores.

As noted previously, the questionnaire also asked whether participants were ever diagnosed with COVID-19 by a medical clinician (COVID-DIAGNOSIS: POS/NEG), whether they had received a positive COVID-19 laboratory test (COVID-TEST: POS/NEG), and whether a family member or close friend had ever experienced serious symptoms or died of COVID-19 (COVID-FAMILY: POS/NEG).

During the analysis, we assumed that the vast majority of the participants who had COVID-19 (either by clinician diagnosis or test) or had a family member affected would experience symptoms during the year preceding survey participation, that is, between March 5, 2020 (1 year prior to the end of the survey) and February 12, 2021 (the day before which a person could be diagnosed with COVID-19 and be expected to have their acute symptoms resolved before the beginning of the survey, 2 weeks later). We justify this assumption as follows. On March 5, 2020, a total of 41.45 cumulative cases of COVID-19 infection were reported in the United States; on February 12, 2021, a total of 27,504,011 cumulative cases were reported; and on March 5, 2021 (the closing of the survey), 28,866,565 cumulative cases were reported [1]. We, therefore, expect that 95% of the participants who experienced acute COVID-19 in the assumed interval were likely resolved at the time of the survey. The remaining 5% may have experienced acute COVID-19 symptoms during the survey period.

### Data Quality Assurance

An initial set of 506 participants was obtained from Gold Research, Inc, based on the following inclusion criteria: age 18 to 70 years, capacity to complete the questionnaire, and enrollment from multiple databases of potential participants.

The subsequent quality assessment used 4 exclusion criteria: (1) participants with the same responses throughout any section of the questionnaire (eg, “1” for all questions), (2) participants indicating they had been diagnosed by a clinician with 10 or more illnesses (data outside depression and anxiety not described here), (3) participants with minimal variance in a picture rating task (all pictures were rated the same or varied only by 1 point; data not described here), and (4) participants reporting inconsistent education level and years of education and participants who completed the questionnaire in less than 500 seconds. After applying the aforementioned exclusion criteria, 74.9% (379/506) of participants qualified for the statistical analysis.

### Statistical Analyses

The aims of this study were divided into 3 categories. First, assuming a similar prevalence of depression and anxiety history among participants with and without COVID-19 history, we asked whether COVID-19 infection (a personal diagnosis or severe symptomology in a close friend or relative) was linked to—and explicitly causative of—increased depression (PHQ-9) or anxiety (state STAI) screening scores. Second, we asked whether COVID-19 infection was linked to—and implicitly causative of—increases in specific-item PHQ-9 scores and specifically in PCS-related scores (in particular, scores corresponding to fatigue, problems with concentration, psychomotor retardation or agitation, and altered appetite) for those patients admitting a COVID-19 diagnosis. Finally, we asked whether COVID-19 infection was linked to increased STB before or after infection and whether any causality in either direction was suggested. In all cases, we considered confounding factors from demographic information, as well as from previous diagnoses of depression and anxiety. Table 3 gives a broad framework of the a priori study design.

**Table 3:** Overview of hypotheses and analyses underlying this study.

Hypothesis^a^	Expected causality^b^	A priori covariates	Analysis tools
COVID-19 diagnosis → total PHQ-9^c^	Explicit	Group A covariates^d^; COVID-19 in relation	Group A analyses^e^
COVID-19 in relation → total PHQ-9	Explicit	Group A covariates; COVID-19 diagnosis	Group A analyses
COVID-19 diagnosis → total STAI^f^	Explicit	Group A covariates; COVID-19 in relation	Group A analyses
COVID-19 in relation → total STAI	Explicit	Group A covariates; COVID-19 diagnosis	Group A analyses
COVID-19 diagnosis → PHQ-9	Implicit	Group A covariates; COVID-19 in relation	Group A analyses
COVID-19 in relation → PHQ-9	Implicit	Group A covariates; COVID-19 diagnosis	Group A analyses
COVID-19 diagnosis ↔ STB^g^ items (3 time windows)	Exploratory	Group A covariates; COVID-19 in relation	Group A analyses; RM-ANOVA^h^
COVID-19 in relation ↔ STB items (3 time windows)	Exploratory	Group A covariates; COVID-19 diagnosis	Group A analyses; RM-ANOVA

^a^Arrows indicate the expected direction of causality (if any).

^b^When expected causality is “explicit,” we possess a baseline measure of the dependent variable, and when “implicit,” causality is believed to be implied by context.

^c^PHQ-9: Patient Health Questionnaire-9.

^d^“Group A covariates” include demographics, depression history, and anxiety history.

^e^“Group A analyses” include Mann-Whitney *U* testing and linear regression covariate analysis.

^f^STAI: State Trait Anxiety Index.

^g^STB: suicidal thoughts and behavior.

^h^RM-ANOVA: repeated-measures ANOVA.

Data analysis was done in 4 parts and numbered 1 to 4 in the following text; the Results section is also organized using this framework. In part 1, we determined differences in participant demographics and depression or anxiety history variables based on their COVID-19 status (ie, COVID-DIAGNOSIS and COVID-FAMILY) to identify potential confounding variables. In part 2, we assessed differences in cumulative PHQ-9 (PHQ9-SUM) and state STAI (STAI-SUM) scores based on COVID-19 status. In part 3, we examined differences in scores for individual PHQ-9 questions (PHQ9-1 through PHQ9-9) based on COVID-19 status. In part 4, we quantified differences in STB (listed in Table 2) scores based on COVID-19 status. Given that STB was also reported for 3 time frames (ie, past 1 year or more, past 1 month to 1 year, and past 1 month), 30 repeated-measures ANOVAs were run (ie, 3 COVID-19 tests × 2 COVID-19 statuses × 5 questions) to assess potential differences between time frames.

For this study, only the COVID-DIAGNOSIS and COVID-FAMILY analyses are reported in the main text. Similar analyses based on a third COVID-19 status variable, positive self-reported COVID-19 laboratory testing (COVID-TEST), appear in Multimedia Appendix 1, given that there was significant overlap between the COVID-DIAGNOSIS and COVID-TEST responses (see the Participant Demographics subsection).

Part 1 specifically involved comparing the medians of demographic variables (reported in the Results section) and 4 depression or anxiety history variables (DEPRESSION-YRS [years carrying depression diagnosis], DEPRESSION-HX [any history of depression diagnosis], ANXIETY-YRS [years carrying anxiety diagnosis], and ANXIETY-HX [any history of anxiety diagnosis] from Table 2) between 2 COVID-19 status groups (ie, COVID-DIAGNOSIS POS vs NEG group; COVID-FAMILY POS vs NEG group) using Mann-Whitney *U* testing (α=.05). *P* values were adjusted for multiple comparisons using Bonferroni correction. Significant differences in median scores (Bonferroni *P*<.05) indicated potential confounders to be controlled in parts 2 to 4.

The analysis for parts 2 to 4 each involved 2 steps. First, the analysis began by comparing the medians of the mental health variables (as grouped in the first paragraph of this section) between COVID-19 status groups by using the Mann-Whitney *U* test (α=.05) with subsequent Bonferroni correction (*P*<.05). Second, to confirm the robustness of these differences to the potential demographic confounders identified in part 1, linear regressions were run to separately model the mental health variables from COVID-DIAGNOSIS and COVID-FAMILY. For each unique pair of mental health (dependent variable) and COVID-19 status variables (independent variable), we built 1 univariate model and 1 multivariate model. The multivariate model included potential confounders identified in part 1 as covariates. To prevent discrepancy between the sample sizes of the POS and NEG groups from causing linear regression models to preferentially fit NEG group data, we applied the synthetic minority oversampling technique (SMOTE) to the POS group data to equalize the number of POS and NEG samples before fitting. SMOTE is considered a standard oversampling technique [40], and we emphasize that it was not implemented until after initial Mann-Whitney *U* testing. For each univariate or multivariate model pair, we compared the crude and adjusted nonstandardized regression coefficients (denoted as B) for the COVID-19 status variable. A difference of >10% was interpreted to indicate confounding and prompted us to report the adjusted regression coefficient [41]; we otherwise reported the crude regression coefficient. Multimedia Appendix 1 provides more details on this approach for assessing potential confounding factors in our main analyses.

Based on our observation that 22.3% (23/103) of the participants in the COVID-FAMILY POS group were also in the COVID-DIAGNOSIS POS group, we included COVID-DIAGNOSIS as a covariate in all adjusted linear regression models involving COVID-FAMILY. However, COVID-FAMILY was not used as a covariate in the models involving COVID-DIAGNOSIS. This decision was based on the observation that 59% (23/39) of the participants in the COVID-DIAGNOSIS POS group were also in the COVID-FAMILY POS group, suggesting that the consequences of a personal COVID-19 diagnosis could not be meaningfully separated from experiencing severe symptoms in a friend or family member.

## Results

### Overview

A small fraction of the participants were members of the COVID-DIAGNOSIS POS (39/379, 10.3%) or COVID-FAMILY POS (28/103, 27.2%) groups (Table 4), which is broadly consistent with reports of illness incidence during the first year of the COVID-19 pandemic (ie, 10%). A total of 23 participants were members of both COVID-DIAGNOSIS POS and COVID-FAMILY POS groups. Chi-square testing indicated higher levels of COVID-DIAGNOSIS POS status in the COVID-FAMILY POS group than in the overall sample (23/103, 22.3%; *P*=.001) and similarly higher levels of COVID-FAMILY status in the COVID-DIAGNOSIS POS group as when compared to the overall sample (23/39, 59%; *P*<.001). Although not presented as a main finding of this paper, these influenced the construction of our regression models, detailed in the Statistical Analyses subsection.

**Table 4:** Participant demographic statistics^a^.

Variable			Sample description					Mann-Whitney *U* test				
			NEG group, median (IQR)		POS group, median (IQR)		*U* statistic			*P* value		Bonferroni *P* value
**COVID-DIAGNOSIS (NEG: n=340; POS: n=39)**												
	Age (years)	45.0 (28.0)		36.0 (19.5)		4644.0			.001		.004	
	Gender	N/A^a^		N/A		5253.0			.007		.03	
	Income	2.0 (3.0)		4.0 (2.0)		4026.5			<.001		<.001	
	Education	3.0 (2.0)		4.0 (3.5)		5315.5			.02		.08	
**COVID-FAMILY (NEG: n=276; POS: n=103)**												
	Age (years)	44.0) (27.25)		39.0 (31.0)		12,976.0			.10		.38	
	Gender	N/A		N/A		13,674.0			.25		>.99	
	Income	3.0 (3.0)		2.0 (2.0)		13,730.0			.30		>.99	
	Education	3.0 (2.0)		3.0 (1.5)		13,661.0			.28		>.99	

^a^N/A: not applicable.

The results are presented according to the 4 parts outlined in the Statistical Analyses section. First, we present the results of testing for differences in demographic and depression or anxiety history values based on COVID-19 status; this analysis was done to identify potential confounding variables for subsequent analyses. These variables were then included in relevant multivariate regression analyses in the second, third, and fourth analysis sections to quantify confounding effects. Second, we determined differences in cumulative depression (PHQ-9) and anxiety (STAI) scores based on COVID-19 status, including univariate and multivariate linear regressions intended to highlight potential confounding effects indicated previously. Third, we performed the same analysis as in part 2 but for each question in the PHQ-9, as opposed to the cumulative score. Fourth, we performed the same analysis as in the previous 2 parts but for STB scores. Note that although this analysis considers the COVID-DIAGNOSIS and COVID-FAMILY variables, an analogous analysis for COVID-TEST is included in Tables S1-S4 in Multimedia Appendix 1. Please refer to Table 2 for descriptions and abbreviations of all the survey questions considered here.

### Analysis of Demographics and Depression or Anxiety History Against COVID-19 Status

This analysis sought to identify covariates for subsequent analyses. Participants in the COVID-DIAGNOSIS POS group reported a lower median age than that reported in the COVID-DIAGNOSIS NEG group (Bonferroni *P*<.05; Table 4), greater median annual household income, and identified as female (gender) more frequently than participants in the NEG group (Bonferroni *P*<.05). Median age, gender, income, and highest education level did not differ between POS and NEG groups for COVID-19 (all Bonferroni *P*s>.05). Although data for the demographic variables ethnicity and employment were also collected, there were <5 samples in most categories for the COVID-DIAGNOSIS POS group, and we were, therefore, unable to perform valid comparisons between the POS and NEG groups for these 2 variables.

Participants in the COVID-DIAGNOSIS POS group did not report a different median DEPRESSION-HX, DEPRESSION-YRS, ANXIETY-HX, or ANXIETY-YRS score than those in the NEG group (all Bonferroni *P*s>.05; Table 5). By contrast, the median DEPRESSION-HX and DEPRESSION-YRS scores were greater among participants in the COVID-FAMILY POS group than those in the NEG group (Bonferroni *P*<.05). The median ANXIETY-HX and ANXIETY-YRS scores were not significantly different between the participants in the COVID-FAMILY POS and NEG groups (all Bonferroni *P*s>.05).

**Table 5:** Separation of major depression and anxiety variables by COVID-19 status.

Variable		Descriptive statistics		Mann-Whitney *U* test				Linear regression confounder analysis			
		NEG group, median (IQR)	POS group, median (IQR)	*U* statistic	*P* value	Bonferroni *P* value	ΔB		B (95% CI)	*P* value	Bonferroni *P* value
**COVID-DIAGNOSIS (NEG: n=340; POS: n=39)**											
	PHQ9-SUM	6.0 (12.0)	13.0 (9.5)	4383.5	<.001	.001	0.25		3.26 (2.17 to 4.36)	<.001	<.001
	STAI-SUM	20.0 (21.0)	29.0 (11.0)	4538.0	.001	.004	0.08		6.64 (4.92 to 8.35)	<.001	<.001
	DEPRESSION-YRS	0.0 (4.0)	1.0 (3.5)	5612.0	.04	.21	—^a^		—	—	—
	ANXIETY-YRS	0.0 (2.0)	0.0 (3.0)	5765.0	.05	.32	—		—	—	—
	DEPRESSION-HX	0.0 (1.0)	0.0 (1.0)	6239.0	.23	>.99	—		—	—	—
	ANXIETY-HX	0.0 (1.0)	0.0 (1.0)	6102.5	.15	.90	—		—	—	—
**COVID-FAMILY (NEG: n=276; POS: n=103)**											
	PHQ9-SUM	5.0 (12.0)	9.0 (12.0)	11,444.5	.002	.01	0.55		0.96 (−0.22 to 2.15)	.11	>.99
	STAI-SUM	20.0 (22.0)	25.0 (18.5)	12,465.5	.03	.20	—		—	—	—
	DEPRESSION-YRS	0.0 (2.0)	0.0 (5.5)	11,800.5	.002	.01	—		—	—	—
	ANXIETY-YRS	0.0 (2.0)	0.0 (4.5)	13,033.0	.07	—	—		—	—	—
	DEPRESSION-HX	0.0 (1.0)	0.0 (1.0)	12,229.0	.004	—	—		—	—	—
	ANXIETY-HX	0.0 (1.0)	0.0 (1.0)	13,195.0	.09	—	—		—	—	—

^a^Regression analysis was not performed given the nonsignificant Mann-Whitney *U* test.

### Analysis of Total Depression and Anxiety Scores Against COVID-19 Status

#### COVID-DIAGNOSIS

The median PHQ9-SUM score was significantly higher in the COVID-DIAGNOSIS POS group than in the NEG group (Bonferroni *P*<.05). The median STAI-SUM score was also significantly higher in the COVID-TEST POS group than in the NEG group (Bonferroni *P*<.05; Table 5). In contrast, when DEPRESSION-HX, DEPRESSION-YRS, ANXIETY-HX, and ANXIETY-YRS were tested for differences between the COVID-DIAGNOSIS POS and NEG groups, no differences were observed (all Bonferroni *P*s>.05; Table 5).

Linear regression modeling of PHQ9-SUM from COVID-DIAGNOSIS with and without age, gender, and income as covariates suggested confounding in the relationship between PHQ9-SUM and COVID-DIAGNOSIS (|ΔB|>0.1); however, the adjusted B coefficient for COVID-DIAGNOSIS was significantly greater than 0 (Bonferroni *P*<.05; adjusted B reported). For the same analysis modeling STAI-SUM from COVID-DIAGNOSIS, we again found confounding (|ΔB|>0.1), but with an adjusted B coefficient for COVID-DIAGNOSIS significantly greater than 0 (Bonferroni *P*<.05; adjusted B reported).

#### COVID-FAMILY

The median PHQ9-SUM score was significantly higher in the COVID-FAMILY POS group than in the NEG group (Bonferroni *P*<.05; Table 5). However, the median STAI-SUM score was not significantly different between the COVID-FAMILY POS and NEG groups (Bonferroni *P*>.05).

The median DEPRESSION-HX and DEPRESSION-YRS scores were significantly greater in the COVID-FAMILY POS group than in the NEG group (Bonferroni *P*<.05). The median ANXIETY-HX and ANXIETY-YRS scores were not significantly different between the COVID-FAMILY POS and NEG groups (Bonferroni *P*>.05).

Linear regression modeling of COVID-FAMILY from PHQ9-SUM with and without DEPRESSION-HX, DEPRESSION-YRS, and COVID-DIAGNOSIS as covariates suggested confounding in the relationship between PHQ9-SUM and COVID-FAMILY (|ΔB|>0.1), with an adjusted B coefficient not significantly more or less than 0 (Bonferroni *P*>.05; adjusted B reported).

### Analysis of Individual PHQ-9 Questions Against COVID-19 Status

#### COVID-DIAGNOSIS

The median scores for PHQ9-4, PHQ9-5, PHQ9-6, PHQ9-7, PHQ9-8, and PHQ9-9 were higher in the COVID-DIAGNOSIS POS group than in the NEG group (Bonferroni *P*<.05; Table 6). The median scores for PHQ9-1, PHQ9-2, and PHQ9-3 were not significantly different between the COVID-DIAGNOSIS POS and NEG groups (Bonferroni *P*>.05).

Linear regressions individually modeling PHQ9-4, PHQ9-5, PHQ9-6, PHQ9-7, PHQ9-8, and PHQ9-9 from COVID-DIAGNOSIS with and without age, gender, and income as covariates suggested that the relationship of COVID-DIAGNOSIS with PHQ9-4, PHQ9-6, and PHQ9-7 was not subject to confounding (|ΔB|<0.1), with crude B coefficients for COVID-DIAGNOSIS being greater than 0 (Bonferroni *P*<.05; crude B reported). The models modeling PHQ9-4 and PHQ9-6 from COVID-DIAGNOSIS suggested confounding based on age, gender, or income (|ΔB|>0.1). However, the adjusted B coefficients for COVID-DIAGNOSIS were significantly greater than 0 (Bonferroni *P*<.05; adjusted B reported).

**Table 6:** Separation of Patient Health Questionnaire-9 (PHQ-9) item variables by COVID-19 status.

Variable		Descriptive statistics		Mann-Whitney *U* test				Linear regression confounder analysis			
		NEG group, median (IQR)	POS group, median (IQR)	*U* statistic	*P* value	Bonferroni *P* value	ΔB		B (95% CI)	*P* value	Bonferroni *P* value
**COVID-DIAGNOSIS (NEG: n=340; POS: n=39)**											
	PHQ9-1	1.0 (1.0)	1.0 (2.0)	5602.5	.05	.41	—^a^		—	—	—
	PHQ9-2	1.0 (2.0)	1.0 (2.0)	5726.5	.07	.62	—		—	—	—
	PHQ9-3	1.0 (2.0)	1.0 (2.0)	6319.5	.31	>.99	—		—	—	—
	PHQ9-4	1.0 (2.0)	2.0 (1.0)	4963.5	.004	.03	0.05		0.46 (0.31 to 0.60)	<.001	<.001
	PHQ9-5	0.0 (2.0)	1.0 (2.0)	5022.0	.004	.03	0.00		0.41 (0.25 to 0.58)	<.001	<.001
	PHQ9-6	0.0 (2.0)	2.0 (1.0)	4049.0	<.001	<.001	0.13		0.78 (0.62 to 0.94)	<.001	<.001
	PHQ9-7	0.0 (1.0)	1.0 (1.5)	4319.0	<.001	<.001	0.19		0.65 (0.50 to 0.80)	<.001	<.001
	PHQ9-8	0.0 (1.0)	1.0 (2.0)	4078.0	<.001	<.001	0.17		0.68 (0.53 to 0.83)	<.001	<.001
	PHQ9-9	0.0 (1.0)	1.0 (2.0)	4904.0	.001	.005	0.21		0.54 (0.39 to 0.70)	<.001	<.001
**COVID-FAMILY (NEG: n=276; POS: n=103)**											
	PHQ9-1	1.0 (1.0)	1.0 (2.0)	13,012.5	.09	.80	—		—	—	—
	PHQ9-2	1.0 (2.0)	1.0 (2.0)	12,291.0	.02	.14	—		—	—	—
	PHQ9-3	1.0 (2.0)	1.0 (2.0)	12,577.0	.04	.32	—		—	—	—
	PHQ9-4	1.0 (2.0)	1.0 (2.0)	12,579.5	.04	.33	—		—	—	—
	PHQ9-5	0.0 (2.0)	1.0 (2.0)	12,315.5	.02	.14	—		—	—	—
	PHQ9-6	0.0 (2.0)	1.0 (2.0)	11,674.0	.002	.02	0.90		0.03 (−0.14 to 0.20)	.75	>.99
	PHQ9-7	0.0 (1.0)	1.0 (2.0)	12,635.5	.03	.31	—		—	—	—
	PHQ9-8	0.0 (1.0)	0.0 (2.0)	11,903.5	.002	.02	1.18		−0.04 (−0.19 to 0.11)	.61	>.99
	PHQ9-9	0.0 (1.0)	0.0 (1.5)	11,671.5	<.001	.004	0.32		0.19 (0.03 to 0.35)	.02	.20

^a^Regression analysis was not performed given the nonsignificant Mann-Whitney *U* test.

#### COVID-FAMILY

The median scores for PHQ9-6, PHQ9-8, and PHQ9-9 were higher in the COVID-FAMILY POS group than in the NEG group (Bonferroni *P*<.05; Table 6). The median scores for PHQ9-1, PHQ9-2, PHQ9-3, PHQ9-4, PHQ9-5, and PHQ9-7 were not significantly different between the COVID-FAMILY POS and NEG groups (Bonferroni *P*>.05).

Linear regressions individually modeling PHQ9-6, PHQ9-8, and PHQ9-9 from COVID-FAMILY with and without DEPRESSION-HX, DEPRESSION-YRS, and COVID-DIAGNOSIS as covariates suggested that the relationships between COVID-FAMILY and all 3 PHQ-9 item scores were subject to confounding (|ΔB|>0.1). Moreover, the adjusted B coefficients for COVID-FAMILY were not significantly more or less than 0 in all the models (Bonferroni *P*>.05; adjusted B reported).

### Analysis of STB Scores Against COVID-19 Status

#### COVID-DIAGNOSIS

This analysis addressed whether the 10 STB questions for the past month (short term, denoted as ST) and between 1 and 12 months ago (midterm, denoted as MT) were greater in the COVID-DIAGNOSIS POS group than in the NEG group. Scores for all STB questions except for S-SAFETY-LT (LT referring to long term) were significantly greater in the COVID-DIAGNOSIS POS group than in the NEG group (Bonferroni *P*<.05; Table 7).

**Table 7:** Separation of suicidal thoughts and behavior variables by COVID-19 status.

Variable			Descriptive statistics					Mann-Whitney *U* test							Linear regression confounder analysis						
			NEG group, median (IQR)		POS group, median (IQR)		*U* statistic			*P* value		Bonferroni *P* value		ΔB			B (95% CI)		*P* value		Bonferroni *P* value
**COVID-DIAGNOSIS (** **NEG: n=340; POS: n=39** **)**																					
	S-PASSIVE-LT	1.0 (1.0)		3.0 (3.0)		3900.0			<.001		<.001		0.28			0.73 (0.53 to 0.94)		<.001		<.001	
	S-PASSIVE-MT	1.0 (1.0)		3.0 (3.0)		3976.0			<.001		<.001		0.40			0.75 (0.54 to 0.95)		<.001		<.001	
	S-PASSIVE-ST	1.0 (1.0)		3.0 (3.0)		4000.0			<.001		<.001		0.16			0.77 (0.57 to 0.97)		<.001		<.001	
	S-ACTIVE-LT	1.0 (0.0)		2.0 (3.0)		4466.5			<.001		<.001		0.39			0.52 (0.31 to 0.72)		<.001		<.001	
	S-ACTIVE-MT	1.0 (0.0)		2.0 (3.0)		4131.5			<.001		<.001		0.22			0.69 (0.48 to 0.89)		<.001		<.001	
	S-ACTIVE-ST	1.0 (0.0)		3.0 (3.0)		3719.5			<.001		<.001		0.29			0.88 (0.67 to 1.09)		<.001		<.001	
	S-PLAN-LT	1.0 (0.0)		2.0 (3.0)		3809.0			<.001		<.001		0.38			0.80 (0.60 to 1.01)		<.001		<.001	
	S-PLAN-MT	1.0 (0.0)		3.0 (3.0)		3950.5			<.001		<.001		0.48			0.68 (0.47 to 0.88)		<.001		<.001	
	S-PLAN-ST	1.0 (0.0)		2.0 (3.0)		4128.0			<.001		<.001		0.60			0.44 (0.25 to 0.63)		<.001		<.001	
	S-HISTORY-LT	1.0 (0.0)		3.0 (3.0)		3832.0			<.001		<.001		0.28			0.76 (0.58 to 0.95)		<.001		<.001	
	S-HISTORY-MT	1.0 (0.0)		3.0 (3.0)		3538.0			<.001		<.001		0.32			0.76 (0.58 to 0.94)		<.001		<.001	
	S-HISTORY-ST	1.0 (0.0)		2.0 (3.0)		4055.0			<.001		<.001		0.28			0.69 (0.50 to 0.87)		<.001		<.001	
	S-SAFETY-LT	1.0 (2.0)		2.0		5449.5			.02		.25		—^a^			—		—		—	
	S-SAFETY-MT	1.0 (2.0)		2.0 (3.0)		5025.0			.002		.03		0.93			0.04 (−0.17 to 0.26)		0.68		>.99	
	S-SAFETY-ST	1.0 (2.0)		2.0 (3.0)		4752.0			<.001		.005		0.81			0.13 (−0.08 to 0.35)		0.22		>.99	
**COVID-FAMILY (** **NEG: n=276; POS: n=103** **)**																					
	S-PASSIVE-LT	1.0 (1.0)		1.0 (2.0)		12,915.5			.05		.77		—			—		—		—	
	S-PASSIVE-MT	1.0- (1.0)		1.0 (2.0)		12,959.5			.06		.85		—			—		—		—	
	S-PASSIVE-ST	1.0 (1.0)		1.0 (2.0)		12,363.0			.008		.13		—			—		—		—	
	S-ACTIVE-LT	1.0 (1.0)		1.0 (2.0)		12,363.0			.008		.13		—			—		—		—	
	S-ACTIVE-MT	1.0 (1.0)		1.0 (1.0)		13,318.0			.11		>.99		—			—		—		—	
	S-ACTIVE-ST	1.0 (0.0)		1.0 (1.0)		12,787.0			.03		.39		—			—		—		—	
	S-PLAN-LT	1.0 (0.0)		1.0 (1.0)		12,990.0			.04		.65		—			—		—		—	
	S-PLAN-MT	1.0 (0.25)		1.0 (2.0)		12,316.5			.006		.09		—			—		—		—	
	S-PLAN-ST	1.0 (0.0)		1.0 (2.0)		12,545.5			.01		.14		—			—		—		—	
	S-HISTORY-LT	1.0 (0.0)		1.0 (1.0)		13,056.5			.05		.74		—			—		—		—	
	S-HISTORY-MT	1.0 (1.0)		1.0 (2.0)		12,622.0			.02		.26		—			—		—		—	
	S-HISTORY-ST	1.0 (0.0)		1.0 (1.0)		12,839.5			.03		.42		—			—		—		—	
	S-SAFETY-LT	1.0 (0.0)		1.0 (0.5)		13,264.5			.08		>.99		—			—		—		—	
	S-SAFETY-MT	1.0 (2.0)		1.0 (2.0)		13,360.0			.15		>.99		—			—		—		—	
	S-SAFETY-ST	1.0 (2.0)		1.0 (2.0)		13,409.0			.16		>.99		—			—		—		—	
	S-PASSIVE-LT	1.0 (2.0)		1.0 (2.0)		12,881.5			.05		.75		—			—		—		—	

^a^Regression analysis was not performed given the nonsignificant Mann-Whitney *U* test.

Linear regressions individually modeling all STB scores, excluding S-SAFETY-LT, from the COVID-DIAGNOSIS group with and without age, gender, and income as covariates suggested that the relationships between COVID-DIAGNOSIS and all 14 scores were confounded (|∆B|>0.1). However, the adjusted B coefficients for COVID-DIAGNOSIS were significantly greater than 0 (Bonferroni *P*<.05; adjusted B reported) in all models except those modeling S-SAFETY-MT and S-SAFETY-ST.

Across time frames (ie, past 1 year or more, past 1 month to 1 year, and past 1 month), significant effects (Bonferroni *P*<.05) were only observed for COVID-DIAGNOSIS NEG participants for having a suicide plan. No other STB variables differed across time frames for participants with a positive or negative COVID-19 diagnosis. It should be noted that similar results were observed for COVID-TEST NEG participants, who also showed differences over time frames in desire for self-harm.

#### COVID-FAMILY

This analysis addressed the same question as COVID-DIAGNOSIS but for COVID-FAMILY. The scores for all STB questions did not significantly differ between the COVID-FAMILY POS and NEG groups (Bonferroni *P*>.05; Table 7). Consequently, no linear regression was performed for these scores.

Across time frames (ie, past 1 year or more, past 1 month to 1 year, and past 1 month), significant effects (Bonferroni *P*<.05) were only observed for COVID-FAMILY NEG participants for having a suicide plan.

## Discussion

### Principal Findings

This study produced 4 sets of findings. One set of findings showed an increase in depression and anxiety scores for individuals with COVID-19 infection history compared to those without, and showed this despite a similar incidence of prior history of depression or anxiety in those with and without COVID-19 history. The second set of findings was that the depression-associated symptoms reported by individuals with COVID-19 history included elevated fatigue, problems with concentration, psychomotor retardation or agitation, altered appetite, feelings of guilt, and elevated suicidality. The first 4 of these symptoms overlap significantly with PCS and raise the hypothesis that the experience of PCS and guilt associated with contracting COVID-19 may drive an increase in total depression-associated symptoms and diagnosis. The third set of findings showed that elevated STB scores in individuals with COVID-19 history preceded COVID-19 diagnosis, suggesting the possibility that participants with greater pre-existing STB were more predisposed to contracting COVID-19. Furthermore, time frame analysis indicated that differences in STB before and during the pandemic were more pronounced in those without COVID-19 history, raising the possibility that the experience of COVID-19 had a greater effect on STB in the uninfected population. Finally, the fourth set of findings showed that an increase in depression-associated symptoms in those with family or friends adversely affected by COVID-19 might correlate with an elevation in prior history of depression. Moreover, the increase in depression symptoms was no greater than expected in the general population. In what follows, each of these findings is discussed in greater detail.

This study found that participants reporting a COVID-19 clinician diagnosis (COVID-DIAGNOSIS POS) tended to produce greater cumulative depression (PHQ9-SUM) and state anxiety (STAI-SUM) scores than those denying a diagnosis of COVID-19 (NEG). Of note, the median cumulative depression score for those reporting COVID-19 fell within a range associated with mild depression (13.0), whereas the median score for those denying COVID-19 fell within a range associated with minimal depression (6.0). We found no significant difference in self-reported history of depression (DEPRESSION-HX and DEPRESSION-YRS) or anxiety disorder (ANXIETY-HX or ANXIETY-YRS) between those with and without COVID-19 history, and no evidence for demographic variables confounding the relationships between cumulative depression scores and COVID-19 infection. This suggested that experiencing COVID-19 was the primary driver of participants’ increased depression- and anxiety-associated symptoms at the time of participation. Although the durations between participants’ diagnoses and survey participation times were not precisely known (however, as noted in the Methods, over 95% of participants were expected to have participated in the questionnaire >2 weeks following infection), the specific symptom profiles related to increases in PHQ-9 scores were consistent with PCS, further suggesting that survey participation occurred after primary COVID-19 infection resolution. This agrees with a previous work that observed increased depression and anxiety scores up to 6 months after infection [21-24].

Five specific depression-associated symptom scores were elevated in those reporting COVID-19 history. One might hypothesize that PHQ9-4, which assesses fatigue; PHQ9-7, which assesses difficulty with concentration; and PHQ9-8, which assesses unusually slow or restless behavior, are directly capturing typical PCS symptoms including fatigue and “brain fog” (or concentration loss) [3,4,27]. PHQ9-5 assesses poor appetite and overeating and may capture anosmia and ageusia associated with PCS. PHQ9-6 assesses feelings of guilt, which may capture feelings of guilt associated with the contraction of COVID-19 [31], as well as some participants’ feelings of guilt in spreading COVID-19 to close relatives. PHQ9-9 assesses suicidal ideation and needs to be interpreted in the context of the broader set of STB questions discussed in the next section. Regarding the clinical significance of the 1- or 2-point differences in the PHQ-9 item score medians between the groups, it is important to note that a 1-point increase corresponds to one-third of the entire symptom scoring interval. A movement from 0 to 1, which was frequently observed, corresponds to a shift from the absence of symptoms to symptoms appearing “several days” over the past 2 weeks. These observations were noted in the absence of significant differences between the groups for history of depression or years of depression. Given the significant statistical findings after correction for multiple comparisons, the detected changes in PHQ-9 item scores are likely to be clinically significant. Overall, the current results around specific depression-associated symptoms are consistent with the hypothesis that the experience of PCS and guilt associated with contracting COVID-19 drive an increase in cumulative depression score and a population shift toward a higher frequency of depression diagnosis among people who contract COVID-19.

A caveat of this analysis is that although PCS-associated symptoms may drive depression scores and, in turn, the expected number of depression diagnoses upward, we cannot state whether depression symptoms associated with PCS contribute to an increase in “true” cases of depression. For instance, whether fatigue associated with PCS is pathophysiologically similar enough to fatigue associated with standard depression to produce similar disease courses and treatment responses is unclear. Although this requires further investigation, we believe that depression is already considered a highly heterogenous diagnosis [42,43]. Moreover, evidence suggests that biomarkers of PCS-associated depression align with those already linked to major depressive disorders [44].

Along with the PHQ9-9 question about suicidal ideation, we assessed 5 STB questions over 3 time windows. We observed that the median scores in the COVID-19–positive group were almost universally greater than those in the COVID-19–negative group. Median scores in the COVID-19–positive group largely suggested STB symptoms were experienced “rarely” or “sometimes,” whereas median scores in the COVID-19–negative group suggested STB symptoms were experienced “never.” Given that many of the STB symptoms assessed (in particular, active ideation, suicide planning, and suicide history) are considered abnormal at any frequency, the statistically significant differences detected herein are likely to be clinically relevant. Most of these differences were robust to potential confounders, with the notable exception of scores corresponding to questions about a suicide safety plan (S-SAFETY-LT, S-SAFETY-MT, and S-SAFETY-ST). Importantly, the differences in median scores for questions inquiring about STB more than 12 months ago—prior to the approximate onset time of the COVID-19 pandemic in the United States and when we expect our participants to have contracted COVID-19—imply that elevated STB scores preceded COVID-19 diagnosis. One possible interpretation is that participants with greater pre-existing STB were more predisposed to contracting COVID-19. This interpretation is consistent with previous work showing that observed STB is associated with a contemporaneous desire for deliberate COVID-19 exposure among the general population [13] and further suggests that elevated STB predating COVID-19 may be associated with actualized contraction of COVID-19. This is also linked to earlier reports that people with increased STB deliberately sought HIV exposure [45-47]. It did not appear that scores for STB questions differed among short-term, midterm, and long-term versions of the same question within those reporting COVID-19 infection, suggesting that the contraction of COVID-19 did not correlate with an increase in suicidality. It should be noted that scores on short-term suicidality questions (inquiring about the past month) remained higher in those reporting COVID-19 infection than in those who did not, which corroborates previous findings that median PHQ9-9 scores were greater in those reporting COVID-19 infection.

This study observed an increase in depression-associated symptoms among those with family or friends adversely affected by COVID-19. Although we found that cumulative depression scores and 3 specific PHQ-9 questions were elevated in these participants (ie, PHQ9-6, PHQ9-8, and PHQ9-9), other analyses suggested that these differences might be explained by depression history variables (eg, DEPRESSION-HX, DEPRESSION-YRS, and COVID-DIAGNOSIS control variables). Thus, no unique relationships could be established between COVID-FAMILY and any other variables studied. Although we are surprised by this finding, we suspect that participants who were emotionally closer to friends or family with COVID-19 were likely to be physically close to them and to be among the 22.3% (23/103) of participants who also became infected with COVID-19. Our results may suggest that people who witnessed more distant relations develop severe or fatal COVID-19 may experience depression, anxiety, and STB at a level equivalent to the portion of the general population that has also been psychologically affected by COVID-19.

### Limitations

The primary limitation of this study is the small sample size: 379 participants of whom 39 (10.3%) reported a COVID-19 diagnosis. As a result, our statistical power was limited, and we were unable to substantively consider confounding the main relationships by using categorical demographic variables reflecting ethnicity and employment. In addition, with respect to our target variables, we could not always distinguish between participants infected with COVID-19 and those with family or friends adversely affected by COVID-19. This resulted from a large population overlap, likely reflecting a general tendency for COVID-19 infection to be shared among close friends or family members. Therefore, it may be fundamentally difficult to separate the personal experience of having COVID-19 from experiencing a friend or close family member with it. Relatedly, it is possible that symptoms of grief may present similarly to symptoms of PHQ-9. Although we did not observe increased PHQ-9 scores among participants who experienced COVID-19 among close friends or family members after adjustment for personal COVID-19 status, we note that any PHQ-9 score elevation in this context may have reflected an increase in grief versus depression.

As a questionnaire-based study, our data are subject to forms of response bias, including acquiescence bias (likely leading to inflated PHQ-9, STAI, and STB scores), social desirability bias (likely leading to deflated PHQ-9, STAI, and STB scores, as well as potential inauthenticity about positive COVID-19 status), and extreme responding (likely leading to extreme PHQ-9, STAI, and STB scores). We expect a degree of this bias to be uncorrelated and therefore be mitigated in our core analyses, which involved internal comparisons of participants with respect to COVID-19 status. Although we warn that the existence of these biases suggests that group-wide medians for PHQ-9, STAI, and STB may be elevated, we note that the same phenomenon would be expected when these tools are deployed in a clinical setting—perhaps to an even greater extent because responses directly influence patient medical management and relationships with providers. Most concerningly, we admit the possibility that correlations between the misreporting of depression, anxiety, or COVID-19 history and biased PHQ-9, STAI, and STB responses may exist that compromise the study’s main finding. Although readers should be mindful of this possibility and seek to corroborate this study’s findings with other studies that do not depend on questionnaires, this possibility reflects a universal weakness of questionnaire-based studies and studies done on a big data scale that cannot easily incorporate laboratory-based experiments.

Other limitations include that the survey participants may have been subject to recall bias and specifically have conflated current STB with past STB. Regarding the question order bias, questions pertaining to depression and anxiety history, COVID-19 history, and PHQ-9/STAI/STB were all separated by more than 10 items. In addition, this study would have benefited from collecting the date of COVID-19 diagnosis or positive test and examining the duration of acute symptoms to accommodate a more precise analysis (eg, consideration of PCS symptomology with respect to illness duration and distance from diagnosis). Relatedly, it is possible that 5% of our COVID-19–positive participants may have been actively subject to acute COVID-19 symptoms. Lastly, possession of occupational information about the participants would have brought more depth to the analyses with regard to the targeted COVID-19 health outcomes.

This study examined US survey participants’ current and recent mental health in association with the change in COVID-19 status between the onset of the COVID-19 pandemic in the United States (approximately March 2020) and the time of data collection (early March 2021). In the context of its limitations, it was found that cumulative depression and anxiety scores were significantly higher in those reporting COVID-19 infection despite a similar prior diagnostic history of depression or anxiety in those with and without COVID-19 infection. The majority of depression-associated symptoms overlapped with those reported for PCS, and reports of increased STB commonly preceded the onset of the pandemic. Where there were STB differences across time frames related to times before and during the pandemic, significant differences were only observed in those who had not had COVID-19 infection. Lastly, increases in depression-associated symptoms were observed in those with family or friends adversely affected by COVID-19, which appeared to be related to an increase in prior history of depression in this group. Altogether, these observations argue that the relationship of COVID-19 with depression or anxiety diagnoses and STB is not obvious and will require a more detailed study along with serial longitudinal assessments.

## Appendix

Multimedia Appendix 1 This is a supplement with additional information about ethical approval, participant recruitment, and statistical methods.

## Abbreviations

PCS: post–COVID-19 syndrome
PHQ-9: Patient Health Questionnaire-9
SMOTE: synthetic minority oversampling technique
STAI: State Trait Anxiety Index
STB: suicidal thoughts and behavior
