# Supplementary material for: Anxiety, Post–COVID-19 Syndrome-Related Depression, and Suicidal Thoughts and Behaviors in COVID-19 Survivors: Cross-sectional Study
Source: JMIR Form Res. 2022 Oct 25;6(10):e36656. doi: 10.2196/36656 (PMC9604174; doi:10.2196/36656)
Supplement: Multimedia Appendix 1 [file formative_v6i10e36656_app1.docx]

# Supplemental Material

## Methods

### Participant recruitment

(Figure S1) provides a visual representation of the lives of completed questionnaires obtained by the authors. We remind the reader that 500-520 completed questionnaires from participants representing an approximate random sampling of U.S. adults were ordered from Gold Research, Inc., and that 509 were ultimately provided.

Supplemental Figure 1. Flow diagram detailing the outcome of the 509 completed questionnaires obtained by the authors from Gold Research, Inc. “QA” refers to the inclusion/exclusion criteria provided in the *Data quality assurance* subsection of Methods.


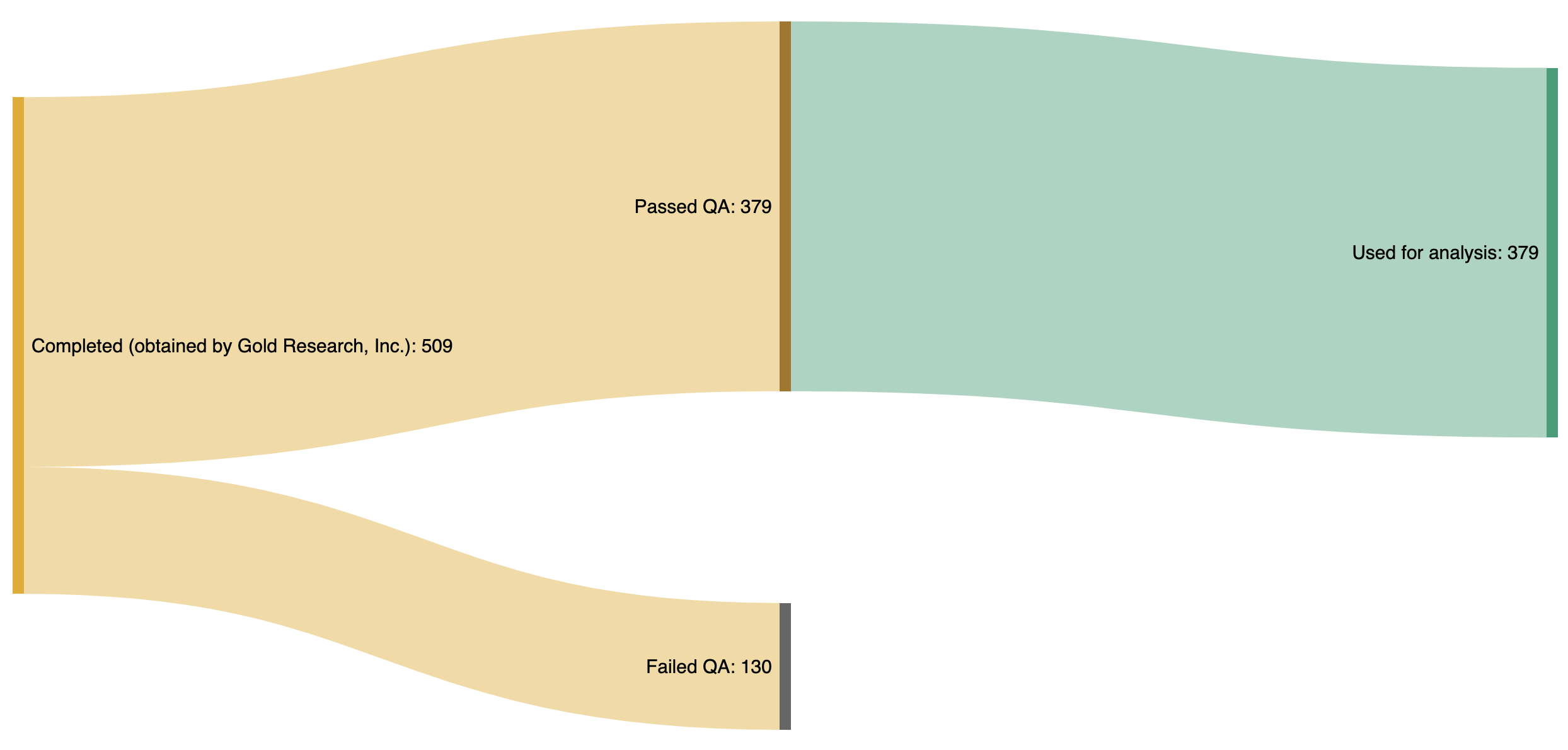


Among other demographic data noted in the main text, we obtained demographic data for the participants, shown graphically in (Figure S2).

Supplemental Figure 2. Heat map representing the distribution of the 379 analyzed participants for state of residence. The numerical scale associated with the color bar denotes the absolute number of participants residing in each state.

**
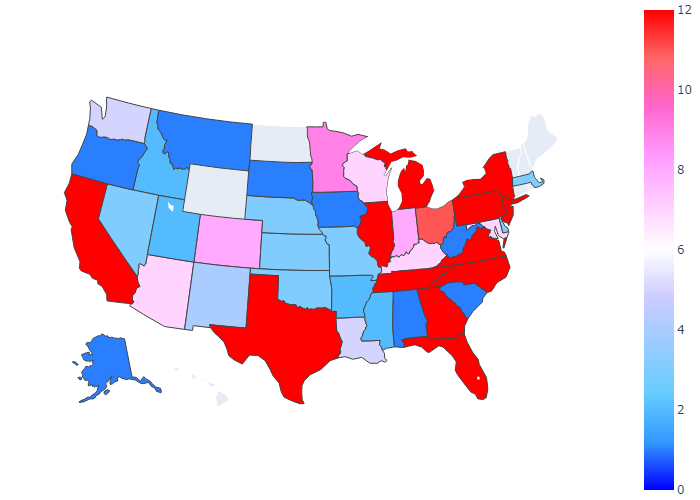
**

### Construction of linear regressions

If, for a particular COVID-19 question, the median of a demographic variable or a depression/anxiety history variable (i.e., DEPRESSION-YRS, DEPRESSION-HX, ANXIETY-YRS, ANXIETY-HX) varied between POS and NEG groups, linear regression was used to assess whether that variable confounded the relationship between COVID status and any statistically significant variable of interest (PHQ9-SUM, STAI-SUM, etc.). For example, if PHQ9-SUM as well as demographic variables AGE and INCOME were found to statistically differ between COVID-TEST POS and NEG groups, we did the following: two linear regressions modeling PHQ9-SUM from COVID-TEST were built, one with and one without AGE and INCOME as covariates. The univariate and multivariate regressions generated crude and adjusted B coefficients (i.e., non-standardized regression coefficients) for PHQ9-SUM, respectively. A difference between adjusted versus crude B coefficients greater than 10% was interpreted to indicate confounding by AGE and/or INCOME (Budtz-Jørgensen et al., 2007). When confounding was present, we reported the adjusted B coefficient and corresponding p-values for the target variable of interest; when not present, we reported the crude B coefficients and corresponding p-values. To prevent the discrepancy between POS and NEG sample sizes from causing the linear regression models to use decision boundaries that neglected POS group data in favor of trivially modeling NEG labels, we applied Synthetic Minority Over-sampling Technique (SMOTE) to the POS group data to equalize the number of POS and NEG samples before fitting. SMOTE is considered a standard oversampling technique that uses interpolation to generate synthetic minority samples (Chawla et al., 2002), and we emphasize that it was not implemented until after the initial Mann-Whitney U testing. Moreover, we ensured that the linear regression B coefficients and p-values obtained after implementing SMOTE did not substantially vary from those obtained without SMOTE (data not included).

## Results

### Analyses for COVID-TEST

Referring to (Table 2), the COVID-TEST variable indicates whether a participant reported ever receiving a positive COVID-19 laboratory test result (POS) or not (NEG).

### Demographics for COVID-TEST

Shown in (Table S1), Mann-Whitney U testing indicated that participants in the COVID-TEST POS group on average reported lower AGE than those in the NEG group (*P_Bonf_* < 0.05). Apart from the differences noted, median AGE, GENDER, INCOME, and Highest Education Level (EDUCATION) were otherwise not found to differ between POS and NEG groups for COVID-TEST. Although data for the categorical demographic variables ETHNICITY and EMPLOYMENT were also collected, we possessed fewer than 5 samples in most categories for the COVID-TEST POS group and were therefore unable to perform valid comparisons of ETHNICITY and EMPLOYMENT distributions between POS and NEG groups.

Supplemental Table 1. Questionnaire items. The first column gives the designations of the scores/variables obtained, the next two columns outline the corresponding questions as phrased on the questionnaire, and the fourth column gives the ranges of possible responses.

|  |  | **Sample Description [NEG, POS]** | | | **Mann-Whitney U Test** | |  |
| --- | --- | --- | --- | --- | --- | --- | --- |
| **Independent Variable** | **Dependent Variable** | **N** | **Median** | **IQR** | **U Statistic** | **p** | **p_Bonf_** |
| COVID-TEST | AGE | [338, 41] | [44.0, 35.0] | [28.0, 21.0] | 4977.5 | .002 | .006 |
|  | GENDER |  | N/A | N/A | 5903.0 | .006 | .02 |
|  | INCOME |  | [2.0, 4.0] | [2.0, 3.0] | 5236.5 | .005 | .02 |
|  | EDUCATION |  | [3.0, 4.0] | [2.0, 3.0] | 5495.0 | .01 | .05 |

### Analysis of total depression and anxiety total scores against COVID-TEST status

(Table S2) addresses whether median PHQ9-SUM and STAI-SUM scores were greater in the COVID-TEST POS group than in the NEG group. Mann-Whitney U test results indicate that median PHQ9-SUM score was significantly greater in the COVID-TEST POS group than in the NEG group (*P_Bonf_* < 0.05). Similarly, they indicate that median STAI-SUM score was significantly greater in the COVID-TEST POS group than in the NEG group (*P_Bonf_* < 0.05).

Regarding whether median DEPESSION-HX, DEPRESSION-YRS, ANXIETY-HX, and ANXIETY-YRS anadiffered between COVID-TEST POS and NEG groups, Mann-Whitney U test results displayed in Table S2 indicate no differences in median DEPRESSION-HX, DEPRESSION-YRS, ANXIETY-HX, and ANXIETY-YRS between COVID-TEST POS and NEG groups (*P_Bonf_* > 0.05).

Linear regressions modeling PHQ9-SUM from COVID-TEST with and without AGE, INCOME, and EDUCATION as covariates suggested confounding in the relationship between PHQ9-SUM and COVID-TEST (|∆B| > 0.1), however the adjusted B coefficient for COVID-TEST was significantly greater than zero (*P_Bonf_* < 0.05, adjusted B reported). For the same analysis modeling STAI-SUM from COVID-TEST, we again found confounding (|∆B| > 0.1) but accompanied by an adjusted B coefficient for COVID-TEST significantly greater than zero (*P_Bonf_* < 0.05, adjusted B reported).

Supplemental Table 2. Separation of major depression and anxiety variables by COVID-19 status. For the various dependent variables split by COVID-TEST (POS/NEG), this table gives basic statistics, Mann-Whitney U test results, and linear regression results including a check for confounding by covariates (see text). The sample sizes for NEG and POS are 338 and 41, respectively.

|  | **Sample Description [NEG, POS]** | | **Mann-Whitney U Test** | |  | **Linear Regression Confounder Analysis** | | |  |  |
| --- | --- | --- | --- | --- | --- | --- | --- | --- | --- | --- |
| **Dependent Variable** | **Median** | **IQR** | **U Statistic** | **p** | **p_Bonf_** | **∆B** | **B*** | **95% CI B*** | **p*** | **p_Bonf_*** |
| PHQ9-SUM | [6.0, 12.0] | [15.0, 21.0] | 4995.5 | .002 | .01 | 0.19 | 2.71 | [1.61, 3.80] | <.001 | <.001 |
| STAI-SUM | [20.0, 29.0] | [21.0, 11.0] | 5050.0 | .002 | .01 | 0.24 | 4.76 | [2.92, 6.60] | <.001 | <.001 |
| DEPRESSION-YRS | [0.0, 1.0] | [3.75, 4.0] | 6014.0 | .06 | .33 |  |  |  |  |  |
| ANXIETY-YRS | [0.0, 0.0] | [2.0, 3.0] | 6249.5 | .11 | .65 |  |  |  |  |  |
| DEPRESSION-HX | [0.0, 0.0] | [1.0, 1.0] | 6275.0 | .11 | .65 |  |  |  |  |  |
| ANXIETY-HX | [0.0, 0.0] | [1.0, 1.0] | 6700.0 | .33 | >.999 |  |  |  |  |  |

### Analysis of individual PHQ-9 questions against COVID-TEST status

(Table S3) addresses whether median scores corresponding to responses to the nine questions composing the PHQ-9 were greater in the COVID-TEST POS group than in the NEG group. Mann-Whitney U testing indicated that median scores for PHQ9-5, PHQ9-6, PHQ9-7, and PHQ9-8 were greater in the COVID-TEST POS group than in the NEG group (*P_Bonf_* < 0.05). median scores for PHQ9-1, PHQ9-2, PHQ9-3, PHQ9-4, and PHQ9-8 were not found to significantly differ between the COVID-TEST POS and NEG groups (*P_Bonf_* > 0.05).

Linear regressions were built to individually model PHQ9-5, PHQ9-6, PHQ9-7, and PHQ9-8 from COVID-TEST with and without AGE, INCOME, and EDUCATION as covariates. Confounding by the covariates was detected in all models (|∆B| > 0.1). However, the adjusted B coefficients for COVID-TEST were significantly greater than zero in all models (*P_Bonf_* < 0.05, adjusted B reported).

Supplemental Table 3. Separation of PHQ9 item variables by COVID-19 status. For the various dependent variables split by COVID-TEST (POS/NEG), this table gives basic statistics, Mann-Whitney U test results, and linear regression results including a check for confounding by covariates (see text). The sample sizes for NEG and POS are 338 and 41, respectively.

|  | **Sample Description [NEG, POS]** | | **Mann-Whitney U Test** | |  | **Linear Regression Confounder Analysis** | | |  |  |
| --- | --- | --- | --- | --- | --- | --- | --- | --- | --- | --- |
| **Dependent Variable** | **Median** | **IQR** | **U Statistic** | **p** | **p_Bonf_** | **∆B** | **B*** | **95% B*** | **p*** | **p_Bonf_*** |
| PHQ9-1 | [1.0, 1.0] | [1.0, 2.0] | 5978.5 | .06 | .57 |  |  |  |  |  |
| PHQ9-2 | [1.0, 1.0] | [2.0, 2.0] | 6015.5 | .07 | .64 |  |  |  |  |  |
| PHQ9-3 | [1.0, 1.0] | [2.0, 2.0] | 6120.5 | .10 | .90 |  |  |  |  |  |
| PHQ9-4 | [1.0, 2.0] | [2.0, 1.0] | 5524.5 | .01 | .12 |  |  |  |  |  |
| PHQ9-5 | [0.0, 1.0] | [2.0, 2.0] | 5181.5 | .002 | .02 | 0.12 | 0.46 | [0.29, 0.62] | <.001 | <.001 |
| PHQ9-6 | [0.0, 2.0] | [2.0, 2.0] | 4817.5 | <0.001 | .003 | 0.22 | 0.45 | [0.29, 0.60] | <.001 | <.001 |
| PHQ9-7 | [0.0, 1.0] | [1.0, 2.0] | 5160.5 | .002 | .02 | 0.25 | 0.30 | [0.16, 0.44] | <.001 | <.001 |
| PHQ9-8 | [0.0, 1.0] | [1.0, 2.0] | 4936.0 | <0.001 | .002 | 0.50 | 0.32 | [0.17, 0.47 ] | <.001 | <.001 |
| PHQ9-9 | [0.0, 0.0] | [1.0, 2.0] | 5632.0 | .008 | .07 |  |  |  |  |  |

### Analysis of individual PHQ-9 questions against COVID-TEST status

(Table S4) addresses whether mean scores for responses to the 10 questions asking about STB within the last month (short-term or ST) and between one month and 12 months ago (mid-term or MT) were greater in the COVID-TEST POS group than in the NEG group. Mann-Whitney U testing indicated that the mean scores for all suicidality/self-harm questions except S-SAFETY-LT were significantly greater in the COVID-TEST POS group than in the NEG group (*P_Bonf_* < 0.05).

Linear regressions individually modeling all STB scores, excluding S-SAFETY-LT, from COVID-TEST with and without AGE, INCOME, and EDUCATION as covariates suggested that the relationships between COVID-TEST and all 14 scores were subject to confounding (|∆B| > 0.1). However, adjusted B coefficients for COVID-TEST were significantly greater than zero (*P_Bonf_* < 0.05, adjusted B reported) in all models except those modeling S-ACTIVE-LT, S-SAFETY-MT, and S-SAFETY-ST.

Supplemental Table 4. Separation of STB variables by COVID-19 status. For the various dependent variables split by COVID-TEST (POS/NEG), this table gives basic statistics, Mann-Whitney U test results, and linear regression results including a check for confounding by covariates (see text). The sample sizes for NEG and POS are 338 and 41, respectively.

|  | **Sample Description [NEG, POS]** | | **Mann-Whitney U Test** | |  | **Linear Regression Confounder Analysis** | | |  |  |
| --- | --- | --- | --- | --- | --- | --- | --- | --- | --- | --- |
| **Dependent Variable** | **Median** | **IQR** | **U Statistic** | **p** | **p_Bonf_** | **∆B** | **B*** | **95% CI B*** | **p*** | **p_Bonf_*** |
| S-PASSIVE-LT | [1.0, 2.0] | [1.0, 3.0] | 4636.0 | <.001 | <.001 | 0.50 | 0.38 | [0.20, 0.56] | <.001 | <.001 |
| S-PASSIVE-MT | [1.0, 2.0] | [1.0, 3.0] | 5014.5 | <.001 | .004 | 0.48 | 0.45 | [0.26, 0.65] | <.001 | <.001 |
| S-PASSIVE-ST | [1.0, 2.0] | [1.0, 3.0] | 4573.5 | <.001 | <.001 | 0.44 | 0.52 | [0.32, 0.71] | <.001 | <.001 |
| S-ACTIVE-LT | [1.0, 1.0] | [0.0, 2.0] | 5470.0 | .002 | .04 | 0.32 | 0.29 | [0.10, 0.47] | .002 | .03 |
| S-ACTIVE-MT | [1.0, 2.0] | [0.0, 3.0] | 4870.5 | <.001 | <.001 | 0.34 | 0.48 | [0.29, 0.67] | <.001 | <.001 |
| S-ACTIVE-ST | [1.0, 2.0] | [0.0, 3.0] | 4360.0 | <.001 | <.001 | 0.32 | 0.66 | [0.46, 0.85] | <.001 | <.001 |
| S-PLAN-LT | [1.0, 2.0] | [0.0, 3.0] | 4298.0 | <.001 | <.001 | 0.30 | 0.78 | [0.59, 0.98] | <.001 | <.001 |
| S-PLAN-MT | [1.0, 3.0] | [0.0, 3.0] | 4164.5 | <.001 | <.001 | 0.30 | 0.80 | [0.60, 1.00] | <.001 | <.001 |
| S-PLAN-ST | [1.0, 2.0] | [0.0, 3.0] | 4693.0 | <.001 | <.001 | 0.50 | 0.38 | [0.21, 0.56] | <.001 | <.001 |
| S-HISTORY-LT | [1.0, 2.0] | [0.0, 2.0] | 4560.5 | <.001 | <.001 | 0.30 | 0.49 | [0.32, 0.66] | <.001 | <.001 |
| S-HISTORY-MT | [1.0, 2.0] | [0.0, 3.0] | 4398.0 | <.001 | <.001 | 0.44 | 0.53 | [0.37, 0.69] | <.001 | <.001 |
| S-HISTORY-ST | [1.0, 2.0] | [0.0, 3.0] | 4492.5 | <.001 | <.001 | 0.44 | 0.48 | [0.31, 0.65] | <.001 | <.001 |
| S-SAFETY-LT | [1.0, 1.0] | [2.0, 2.0] | 6580.5 | .27 | >.999 |  |  |  |  |  |
| S-SAFETY-MT | [1.0, 1.0] | [2.0, 3.0] | 5939.0 | .04 | .60 | 1.00 | 0.00 | [-0.21, 0.21] | >.999 | >.999 |
| S-SAFETY-ST | [1.0, 2.0] | [2.0, 3.0] | 5486.5 | .005 | .08 | 0.80 | 0.11 | [-0.11, 0.33] | .32 | >.999 |
